# Macular pigment-enriched oil production from genome-edited microalgae

**DOI:** 10.1186/s12934-021-01736-7

**Published:** 2022-02-19

**Authors:** Inhwa Song, Sunbin Kim, Jongrae Kim, Hyeonjun Oh, Junhwan Jang, Su Jin Jeong, Kwangryul Baek, Weon-Sun Shin, Sang Jun Sim, EonSeon Jin

**Affiliations:** 1 Department of Life Science, Research Institute for Natural Sciences, Hanyang University, Seoul, 04763 Republic of Korea; 2 Department of Food and Nutrition, College of Human Ecology, Hanyang University, Seoul, 04763 Republic of Korea; 3 Department of Chemical and Biological Engineering, Korea University, Seoul, South Korea

**Keywords:** CRISPR-Cas9, *Chlamydomonas reinhardtii*, Macular pigment-enriched oil, Zeaxanthin epoxidase, ADP-glucose pyrophosphorylase

## Abstract

**Background:**

The photosynthetic microorganism *Chlamydomonas reinhardtii* has been approved as generally recognized as safe (GRAS) recently, this can excessively produce carotenoid pigments and fatty acids. Zeaxanthin epoxidase (*ZEP*), which converts zeaxanthin to violaxanthin, and ADP-glucose pyrophosphorylase (*AGP*). These are key regulating genes for the xanthophyll and starch pathways in *C. reinhardtii* respectively. In this study, to produce macular pigment-enriched microalgal oil, we attempted to edit the *AGP* gene as an additional knock-out target in the *zep* mutant as a parental strain.

**Results:**

Using a sequential CRISPR-Cas9 RNP-mediated knock-out method, we generated double knock-out mutants (dZAs), in which both the *ZEP* and *AGP* genes were deleted. In *dZA1*, lutein (2.93 ± 0.22 mg g^−1^ DCW: dried cell weight), zeaxanthin (3.12 ± 0.30 mg g^−1^ DCW), and lipids (450.09 ± 25.48 mg g^−1^ DCW) were highly accumulated in N-deprivation condition. Optimization of the culture medium and process made it possible to produce pigments and oil via one-step cultivation. This optimization process enabled dZAs to achieve 81% higher oil productivity along with similar macular pigment productivity, than the conventional two-step process. The hexane/isopropanol extraction method was developed for the use of macular pigment-enriched microalgal oil for food. As a result, 196 ± 20.1 mg g^−1^ DCW of edible microalgal oil containing 8.42 ± 0.92 mg g^−1^ lutein of oil and 7.69 ± 1.03 mg g^−1^ zeaxanthin of oil was produced.

**Conclusion:**

Our research showed that lipids and pigments are simultaneously induced in the dZA strain. Since dZAs are generated by introducing pre-assembled sgRNA and Cas9-protein into cells, antibiotic resistance genes or selective markers are not inserted into the genome of dZA, which is advantageous for applying dZA mutant to food. Therefore, the enriched macular pigment oil extracted from improved strains (dZAs) can be further applied to various food products and nutraceuticals.

**Supplementary Information:**

The online version contains supplementary material available at 10.1186/s12934-021-01736-7.

## Background

Microalgae have substantial potential to produce natural compounds such as fatty acids and carotenoid pigments [1, 2]. In recent decades, microalgae research has focused on the utilization of these natural compounds from a commercial perspective. In addition, genetic engineering enables the production of value-added industrial compounds [3–5]. Improved extraction methods from microalgal biomass can accelerate the commercialization of microalgae [6, 7]. In the past decade, biofuel production from microalgal biomass has been developed, however, the low productivity of biofuels from microalgae has hampered commercial viability [7]. Thus, in addition to economically unviable biofuel production, microalgal oil has recently been considered an alternative to vegetable oil as a food additive [8, 9]. Research on microalgal oil is currently focused on the discovery of strains with appropriate fatty acid content for edible oil. In addition, oil extraction method suitable for microalgae, and the process of refining oil to meet the quality standards of food additives are also being developed [8].

The model green microalgae *Chlamydomonas reinhardtii* has recently been re-evaluated as an industrial strain, which can be made commercially available [10]. Furthermore, it was recently registered as GRAS (Notice No. GRN 000773), which made *C. reinhardtii* commercially viable. An attempted strategy for the commercialization of *C. reinhardtii* is to enable high lipid production or high value-added pigment production. This is because fatty acid and carotenoids biosynthesis are well defined [11].

Previous studies have shown the possibility of producing microalgal oil from *C. reinhardtii* [12, 13]. The most preferred strain for this is the starchless *C. reinhardtii,* which accumulated twice as much fatty acid as the wild type [14]. The starchless phenomenon is caused by the knockout of ADP-glucose pyrophosphorylase (*AGP*). This strategy has been reported to increase fatty acid synthesis, which is a competitive pathway for starch synthesis [15, 16]. This competitive metabolic regulation between fatty acids and starch is the same as that in *Arabidopsis* [17]. In addition, overexpression of diacylglycerol acyltransferase Type2 [18] or knockdown of diacylglyceryl-N-trimethyl homoserine synthesis protein [19] in the glycerolipid synthesis pathway was attempted to increase triacylglycerol (TAG) accumulation in C. reinhardtii. Recently, it has been reported that fatty acid production is increased with an increase in the precursor pool of fatty acids such as pyruvate or acetyl-CoA [20, 21]. The metabolic pathway involved in the biosynthesis of carotenoid pigments has been elucidated in most processes [22]. The level of zeaxanthin is regulated through the interconversion to violaxanthin by zeaxanthin epoxidase (ZEP) and violaxanthin de-epoxidase (VDE) [23]. In the conversion cycle, it is reported that due to the loss of function of the *ZEP* gene, zeaxanthin was accumulated in *C. reinhardtii*. [23, 24]. Zeaxanthin is widely distributed, along with lutein, in the macular region of the retina and the lens of the human eye and is particularly highly concentrated in the central part of the macula [25, 26]. Lutein and zeaxanthin are called macular pigments in clinical practice, as they are essential pigments in maintaining human eye health. Indeed, many studies have reported the relationship between macular pigments and eye-related disorders [27–30]. Therefore, macular pigments are considered effective agents for lowering the incidence of age-related macular degeneration (AMD), which is highly associated with impaired vision and blindness, and cataracts in the elderly [26].

However, macular pigments are not synthesized naturally in the body of humans and animals; thus, the content of these pigments seems to decrease continuously from late twenties [30]. For this reason, an additional supply of zeaxanthin is beneficial because the intake of zeaxanthin from vegetables in a normal diet is usually insufficient [31]. *C. reinhardtii* could be the additional source of zeaxanthin and a strategy for the accumulation of excess zeaxanthin in *C. reinhardtii* has been reported in a previous study [24]. The strategic regulation of well-known biosynthetic pathways offers us significant opportunities to improve *C. reinhardtii*. In this study, a strategy to simultaneously regulate both biosynthesis pathways of fatty acids and xanthophylls was established to produce macular pigment-enriched microalgal oil. This algal oil can be used as a functional food oil to help in preventing chronic eye disease without taking an ocular formula. Using the CRISPR-Cas9 mediated knockout method, deletion of the AGP gene was induced in the *ZEP* knockout strain, which was generated in a previous study [24]. Based on the two distinct phenotypic characteristics of zeaxanthin accumulation and the lack of starch, the creation of the mutants was confirmed. In addition, the medium composition was modified to simplify the process of inducing TAG accumulation to increase the commercial viability of the mutant strain. Through these efforts, we developed a commercially applicable strain that produces macular pigment-enriched microalgal oil under optimized culture conditions.

## Results and discussion

### Generation of the macular pigment-enriched microalgal oil-accumulating mutant by CRISPR-Cas9

**Fig. 1:**
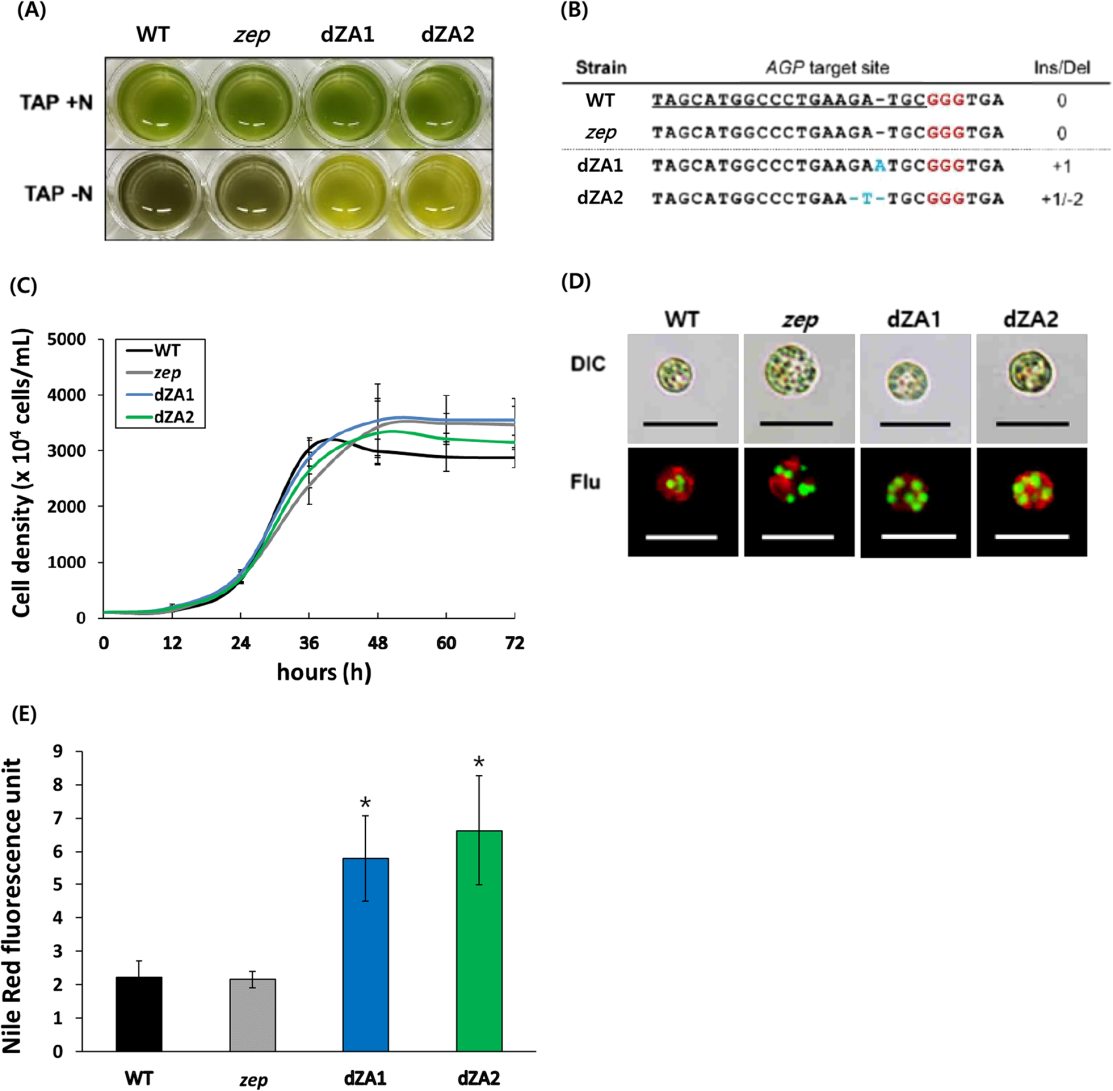
Morphological features and genomic sequence analysis of *AGP* knock-out mutants (dZAs). **A** Analysis of starch phenotype by iodine staining. After being cultured in the nitrogen-starvation medium, a cell was stained with iodine solution, and the dark green color indicates the presence of starch as in the lower panel. **B** Genomic DNA sequence alignment of *AGP* of the wild type, *zep* line, dZA1, and dZA2. The 20-bp target sequence is underlined, and the red character is the PAM sequence. Blue characters indicate the insertion or deletion sequence on the *AGP* site in the dZAs. **C** Cell growth of the dZA1 and dZA2 under normal conditions was compared to that of the wild type and *zep* line. The data shown are representative of three independent experiments (n  > 9). Error bars indicate standard deviation. **D** Triacylglycerol (TAG) accumulation in the cells cultured in the nitrogen-starvation medium is visualized by BODIPY staining. The green dot indicates the oil droplet in the cytosol, and the red color indicates the chloroplast. The size bar represents 10 µm. **E** The fluorescence intensity of the cells cultured under nitrogen-starvation conditions after Nile red-staining. The data shown are representative of three independent experiments (n  > 3)

In a previous report, zeaxanthin-accumulating *C. reinhardtii* (*zep*) was generated by CRISPR-Cas9-mediated gene editing [24]. The wild type of *C. reinhardtii* can only accumulate zeaxanthin under high light stress. However, *zep* can accumulate zeaxanthin even under normal conditions without high light stress. Using the properties of the *zep* strain, we attempted to produce microalgal oil containing a high content of macular pigments. Given that ADP-glucose pyrophosphorylase (AGP) is one of the key regulatory enzymes in the production of lipids (oils) in *C. reinhardtii*, the AGP gene was selected as a gene-editing target of the *zep* line by CRISPR-Cas9. Because a mutation in the AGP gene was created without the insertion of any antibiotic resistance gene, it was difficult to select the correct mutants from many transformants by PCR. Therefore, mutant screening was conducted based on the limitation of starch synthesis through the reduction of the precursor in the AGP knockout mutant [16]. As a result, the AGP knockout mutants (dZAs) were selected visually based on starchless features (Fig. 1A). The dark green color of the wild type and *zep* line indicated the presence of starch, whereas starch was absent in dZA mutants (dZAs). Analysis of the genome sequence revealed Ins/Del mutations in the target sequence in the two dZA1 and 2 (Fig. 1B). DNA insertion or deletion at the RuvC site (between  + 17 and  + 18 position on the target site) in dZAs showed that the gene targeted by CRISPR-Cas9 was precisely edited.

Since dZAs generated serial mutations, we determined the effect of the *AGP* mutation on the growth of dZAs using growth analysis and microscopic fluorescence analysis. The growth patterns of dZA1 and dZA2 were almost identical to those of the parental *zep* line and wild type (Fig. 1C). This indicates that the edited lesions of the AGP gene in dZAs does not have any detrimental effect on the growth of dZAs and increases the oil content. To validate the higher TAG accumulation in the dZAs, oil droplets in the cells were visualized through microscopic fluorescence analysis and compared with the parental strains (Fig. 1D). Further, for the quantification of triacylglycerol (TAG), we measured the Nile red fluorescence of each cell line growing under nitrogen starvation conditions and normalized this to the same cell numbers. The two dZA strains displayed more than two-fold higher Nile red fluorescence than the parental strains (Fig. 1E). These results showed that the dZAs exhibited increased TAG synthesis through metabolite changes when AGP was knocked out.

### Accumulation of macular pigments and lipid in the dZAs

**Fig. 2:**
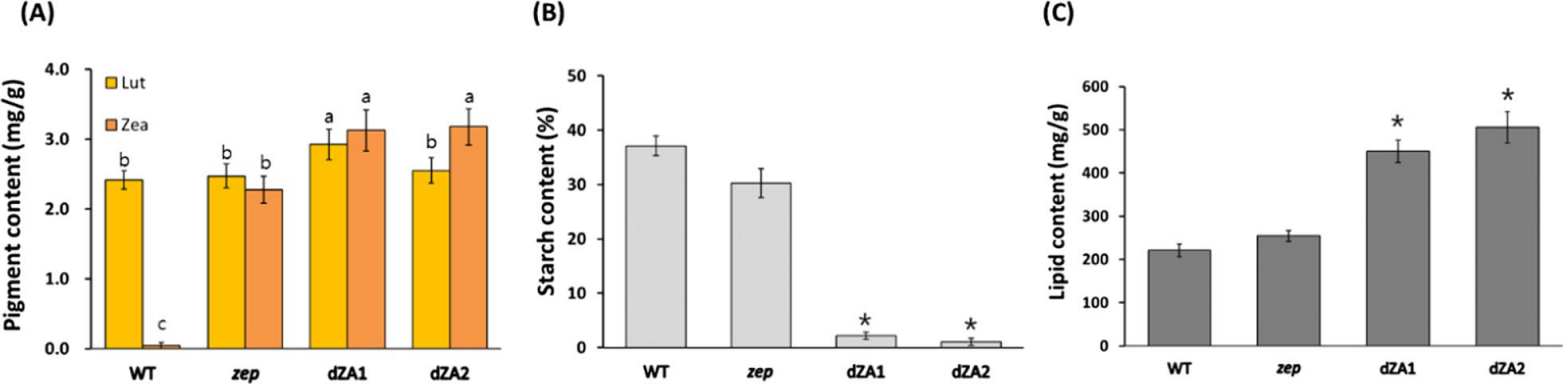
Comparative and quantitative analysis of pigments, starch, and lipid of cells cultured under nitrogen-starvation medium (TAP-N). **A** Pigment contents in the wild type and mutants measured by high performance liquid chromatography (HPLC). Lut lutein; Zea zeaxanthin. **B** Starch content in the wild type and mutants measured by the enzymatic hydrolysis method. **C** Total lipid content in the wild type and mutants measured by the chloroform/methanol extraction method. The data shown are representative of three independent experiments (n  > 9). Error bars indicate standard deviation. Asterisks indicate statistically significant differences based on Student’s t test (P  < 0.05). ^a−c^In a row without a common superscript letter, differ (*P*  < 0.05) as analyzed by two-way ANOVA and the Tukey test. (n  = 6)

We quantitatively analyzed and compared metabolites such as pigments, starch, and lipids in the dZA1 and 2 (dZAs) with those in the parental strains. Because the dZAs were generated using *zep* as the parental strain, they concurrently accumulated lutein and zeaxanthin (macular pigments). First, the content of pigments in the dZAs was analyzed using high-performance liquid chromatography (HPLC). The dZAs had both higher lutein (2.93 or 2.55 mg g^−1^ DCW) and zeaxanthin (3.12 or 3.18 mg g^−1^ DCW) content than those of the *zep* line, where lutein and zeaxanthin content is 2.47 and 2.27 mg g^−1^ DCW, respectively (Fig. 2A). Interestingly, the macular pigments in the dZAs were observed to be higher than that in *zep*. Approximately 35% higher zeaxanthin was present in both dZAs than that of parental line. In addition, dZA1 showed approximately 20% higher lutein content than the WT or *zep* line. However, the lutein content of dZA2 was almost the same as that of the WT or *zep* strain under N-deprivation culture conditions. The reason for the different lutein contents in dZA1 and dZA2 is not known. The metabolite changes such as the remaining D-glucose-1-phosphate and ATP in the dZAs could directly affect the associated carbon metabolic circuits. As one of these effects, carotenogenesis might increase in dZAs [11, 32]. Similar metabolite changes have been reported in citrus plant, indicating that decreased starch synthesis is associated with increased carotenoid levels [33].

To quantify starch synthesis, dried biomass was hydrolysed using an amyloglucosidase/α-amylase combination method, and the starch was quantified by the amount of dissociated glucose as explained in the Materials and Methods section. The starch content based on a DCW was approximately 35% in the wild-type and *zep* line, and approximately 2% in the dZAs (Fig. 2B), which was consistent with the results of iodine staining (Fig. 1A). Total lipid analysis showed an increase in lipid content in the dZAs with 450.0 mg g^−1^ DCW in dZA1 and 505.6 mg g^−1^ DCW in dZA2 (Fig. 2C). Whereas, the total lipid content of the wild type and *zep* was 221.3 mg g^−1^ DCW and 254.3 mg g^−1^ DCW, respectively (Fig. 2C). Such enhancements of lipid content in dZAs indicated that carbon flux to starch production had been changed to fatty acid biosynthesis owing to the defective starch biosynthesis shown in AGP knockout mutants (dZAs) under N-starvation conditions.

**Fig. 3:**
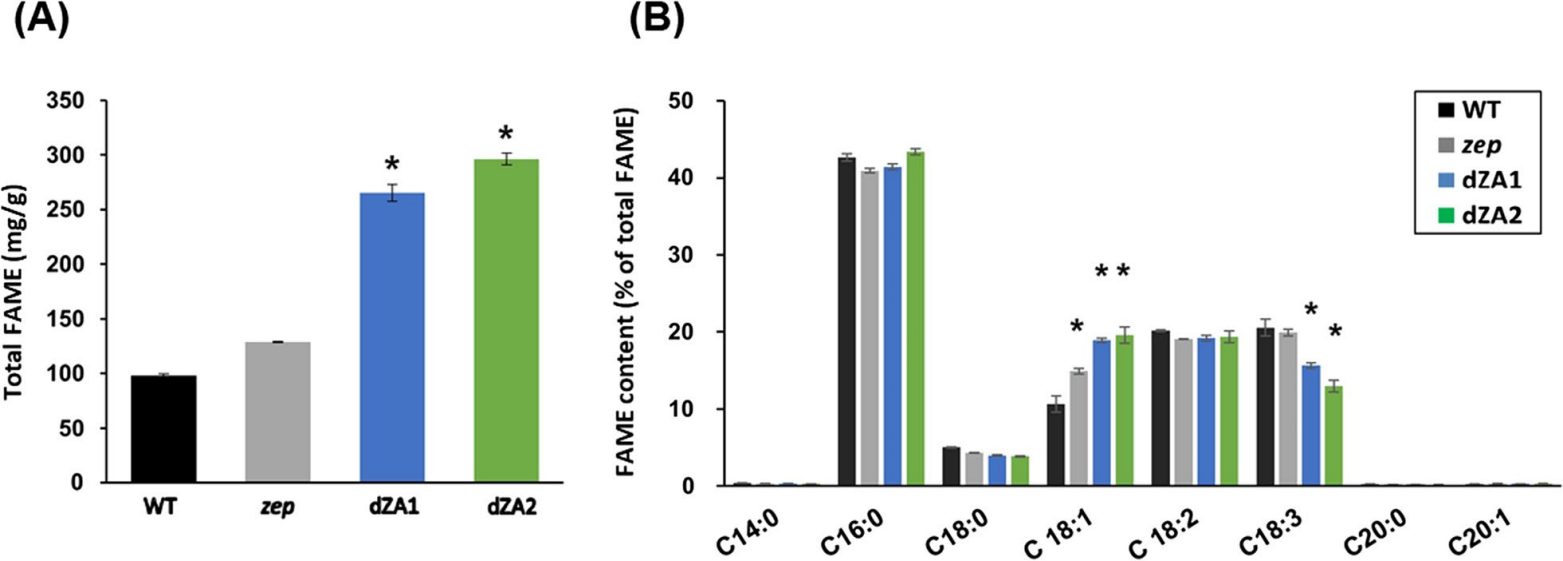
Analysis of fatty acids of cells cultured in nitrogen-starvation medium (TAP-N). **A** Amount of total FAMEs. **B** The profile of fatty acid methyl esters (FAME). FAME was analyzed by gas chromatography. Five major species of fatty acids were detected, and C14 and C20 were negligible. All experiments were conducted in triplicate. Asterisks indicate statistically significant differences by Student’s t test (P  < 0.05)

Fatty acids were also analyzed by gas chromatography (GC-FID) to compare the fatty acid methyl ester (FAME) content and the composition of each strain. After cultivation under TAG-inducing conditions, the content of total FAME measured by GC-FID was approximately half of the amount of total lipids extracted by the Bligh and Dyer method (Fig. 2C), which gave a result of 265 mg g^−1^ DCW in dZA1 and 296 mg g^−1^ DCW in dZA2, whereas it was 98 mg g^−1^ DCW and 128 mg g^−1^ DCW in the wild type and mutant (*zep*), respectively (Fig. 3A). According to these results, the fatty acid composition of the dZA strains was highly similar (Fig. 3B). Because the knockout of *AGP* caused the transfer of precursors from starch synthesis to lipid biosynthesis, the composition of fatty acids should be in accordance with the metabolic pathway of fatty acid synthesis. However, C18:1 increased from 10.9% in the wild type to 19.2–19.9% in the dZAs. Simultaneously, C18:3 decreased as C18:1 increased in the dZAs. This unexpected phenomenon in the desaturation of fatty acids in starchless *C. reinhardtii* has been reported in a previous study [34]; however, there are no clearly identified mechanisms for this to date. Additionally, the minor fatty acid species, C14 and C20, were not found to be the same as those in a previous report [34, 35].

### Improvement of culture processes for lipid production

**Fig. 4:**
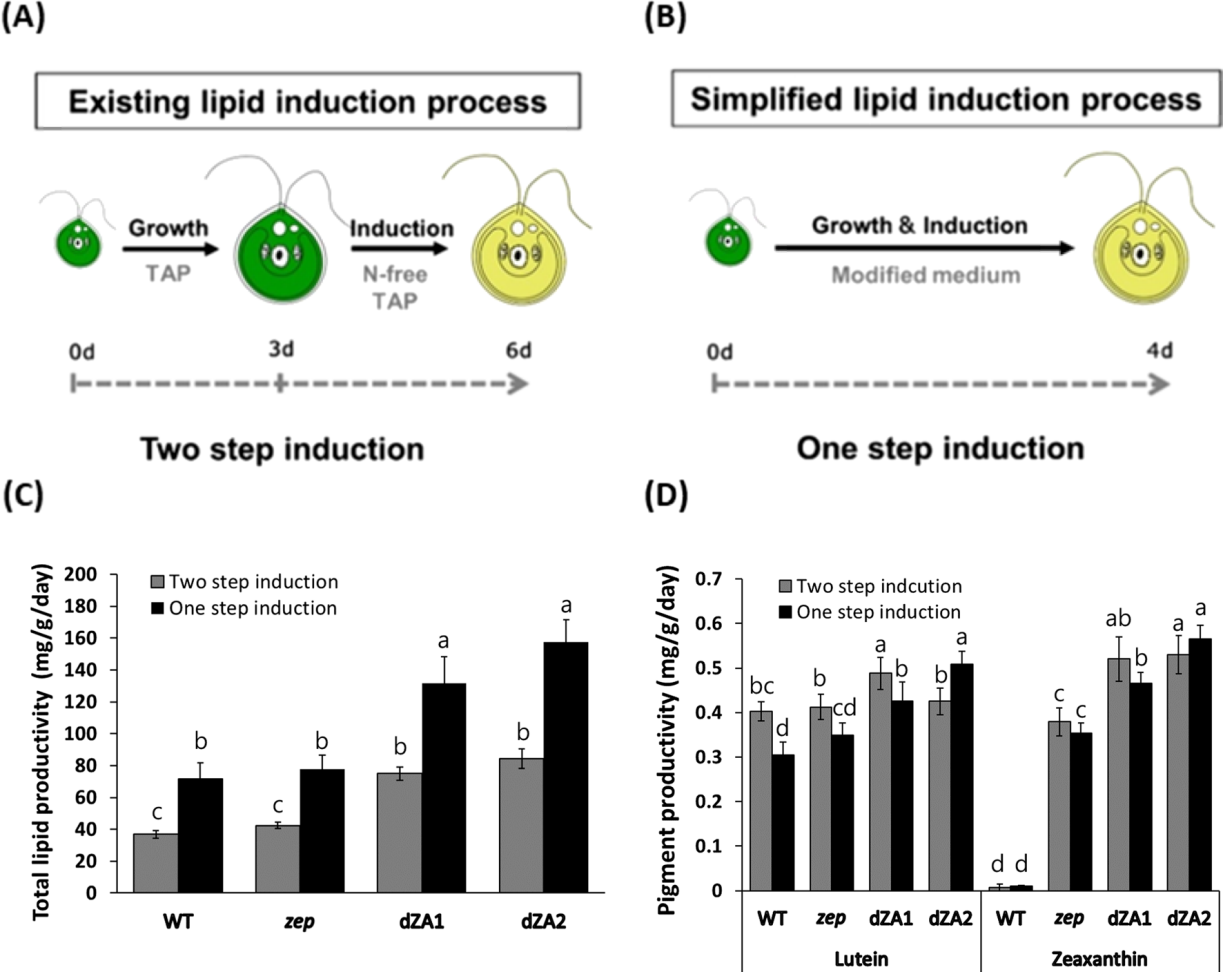
Strategic modification of the cultivation process and optimization of culture medium. **A** Diagram of existing oil-induction process called the two-step induction process. This process includes a medium change step, and the duration of the entire process is 6 days. **B** Diagram of simplified oil-induction process called the one-step induction process. The duration of the oil induction process was reduced by the lack of the medium change process by using a modified culture medium (1/4NP2A medium). **C** Comparison of lipid productivity between the two lipid-induction processes. **D** Comparison of pigment productivity between the two oil induction processes. The data shown are representative of three independent experiments (n  = 9). ^a−c^In a row without a common superscript letter, differ (*P*  < 0.05) as analyzed by two-way ANOVA and the Tukey test (n = 6)

The general process for oil production by *C. reinhardtii* was separated into two steps: cell growth and oil induction. Nitrogen-free medium is normally required for TAG induction after growth, which indicates that the process of harvesting cells and changing the medium is required (Fig. 4A). However, this process is time-consuming and not cost-effective for large-scale cultivation. Therefore, we simplified the oil-induction process by changing the composition of nutrients in the Tris–acetate-phosphate (TAP) medium (Fig. 4B). To determine the optimal design for culture medium, nitrogen, phosphorus, and acetate, which are the main components of TAP medium, were tested by the combination of various concentrations of nitrogen and phosphate ranging 1/8–one-fold, and acetate ranging ½–two-fold in TAP, respectively. As a result, the combination of 1/4 N and 1/4 P with two-fold acetate containing TAP medium revealed the best induction of oil with the desirable amount of macular pigment contents, we called this medium as 1/4NP2A medium. The 1/4NP2A medium included 1.875 mM NH_4_Cl, 0.155 mM K_2_HPO_4_, 0.103 mM KH_2_PO_4_, and 2 mL L^–1^ glacial acetic acid, and others are the same as the TAP medium.

The culture method using this medium was called one-step oil induction process (one-step process), in which cell growth and oil induction occurred in the same medium. Lipid and pigment production from dZA1 were compared between one-step process and the conventional two-step process. Cells growing in the one-step culture condition reached the stationary phase slowly, and the final cell density at 4 days in one-step culture was almost 2/3 than in the normal media (Fig. 1C; Additional file 1: Fig. S1A). In contrast, cells in a two-step process reached the stationary phase fast at normal medium but maintained the cell density during the N-deprivation for 3 more days (Additional file 1: Fig. S1B). Interestingly, at the end of cultivation, the lipid content of dZA1 and dZA2 in the one-step process was enhanced by 1.6-fold and 1.9-fold, respectively, compared to that in the parental strain (*zep*), based on dry weight (Additional file 1: Table S1). However, when we compared the lipid productivity of dZA strains between one-step and two-step processes, the average value of lipid productivity (144.51 mg/g/day) for dZAs in a one-step process was 1.8-fold that of the two-step process (79.64 mg/g/day) (Fig. 4C).

Pigment production by dZA mutants under oil induction conditions was compared with that of the parental line (*zep*). The average value of the macular pigment content of dZAs in the one-step process was 38% higher than that of the parental line (*zep*), but this value in the two-step process was 23% higher than that of *zep* (Additional file 1: Table S1). Further, the macular pigment productivity of dZAs between the two processes was compared, and the average value of pigment productivity in the one-step process was slightly higher or at least the same as that in the two-step process (Additional file 1: Table S1; Fig. 4D).

A one-step process consisting of reduced nitrogen and phosphate provided by the 1/4NP2A medium resulted in slower cell growth compared to that in normal medium (Fig. 1C and Additional file 1: Fig. S1A). However, for the production of oil and pigment together, this process can shorten the culture time by approximately 33% compared to that with the N-starvation process, and medium replacement is not required; thus, without losing pigments, this one-step process enables the production of pigment-enriched microalgae oil for possible food applications.

### Optimization of lipid and pigment extraction for edible use

**Fig. 5:**
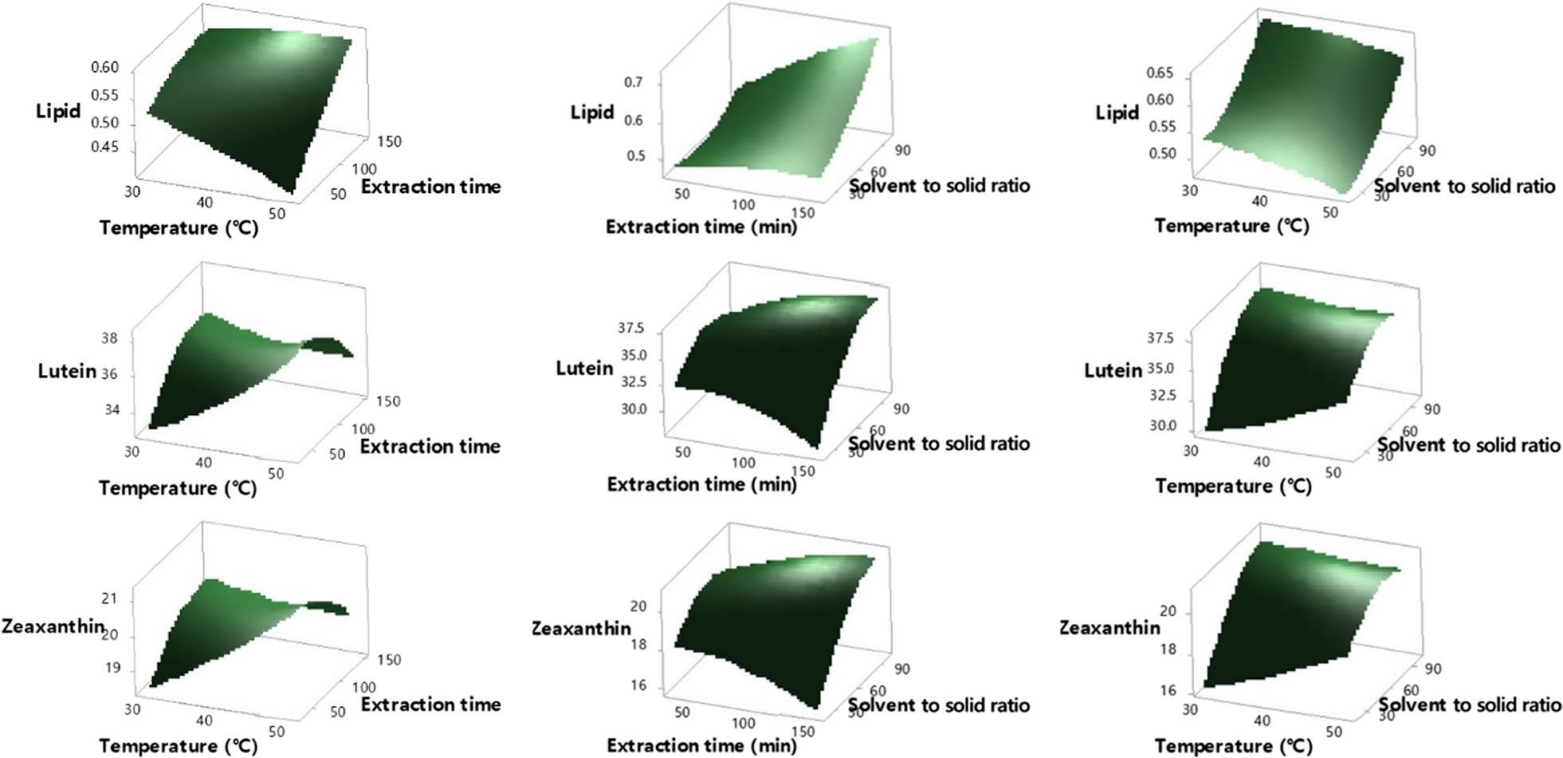
Response surface plot of lipids, lutein, and zeaxanthin. Extraction temperature, extraction time, and solvent-to-biomass ratio were comparatively analyzed. All analyzes were performed in triplicate (n  = 3)

For efficient oil extraction, a mixture of aqueous and organic solvents is the most feasible system [8]. The cell membrane and lipid binding are disrupted by the aqueous solvent, and the lipids can sufficiently diffuse from the cell to the organic solvent. A mixture of chloroform and methanol is generally used for lipid extraction from microalgae, as in the Bligh and Dyer method. However, this mixture is not suitable for edible oil extraction because of the toxicity of chloroform. Therefore, a method of extracting microalgal oil using a solvent that complies with the food additive regulations (Q3C) [36] had to be developed. In this study, we optimized the extraction method using HI solution (hexane/isopropanol  = 3:2, v/v) following Hara and Radin [37] as a solvent for the edible use of pigment-enriched microalgal oil. A Box–Behnken design was used for a response surface methodology [38] with a total of 15 runs, and three replicates at the central point were performed using Minitab 18. The three variables, temperature (X_1_, 30–50 °C), time (X_2_, 30–150 min), and the solvent/biomass ratio (X_3_, 20 to 100 mL/g) were tested along with pigment extraction yield and lipid extraction yield as responses (Fig. 5). The variability of pigments and lipids could be explained by a second-order polynomial equation with three variables (R^2^ values ranged from 0.83 to 0.92). The formulae are as follows:

$$\begin{aligned} {\text{Lipid}} & = 0.725 - 0.0029X_{1} - 0.00165X_{2} - 0.00316X_{3} - 0.000067X_{1} \times X_{1} - 0.000004X_{2} \times X_{2} \\ & + 0.000019X_{3} \times X_{3} + 0.000054X_{1} \times X_{2} + 0.000019X_{1} \times X_{3} + 0.000019X_{2} \times X_{3} \\ \end{aligned}$$

$$\begin{aligned} Lutein =\, & 15.4 + 0.167X_{1} + 0.1237X_{2} + 0.2788X_{3} + 0.00434X_{1} \times X_{1} - 0.000366X_{2} \times X_{2} \\ & - 0.001491X_{3} \times X_{3} - 0.00267X_{1} \times X_{2} - 0.00289X_{1} \times X_{3} + 0.000821X_{2} \times X_{3} \\ \end{aligned}$$

$$\begin{aligned} {\text{Zeaxanthin}} & = 7.02 + 0.19X_{1} + 0.0491X_{2} + 0.1610X_{3} + 0.00118X_{1} \times X_{1} - 0.000130X_{2} \times X_{2} \\ & - 0.000758X_{3} \times X_{3} - 0.001342X_{1} \times X_{2} - 0.00187X_{1} \times X_{3} + 0.000444X_{2} \times X_{3} \\ \end{aligned}$$

The linear term for time and ratio for lipids, the linear and quadratic terms for the ratio, the interaction ratio with time for lutein, and the linear term for the ratio for zeaxanthin were significant. The p value for the lack-of-fit for the three dependent variables was much higher than 0.05, which indicated that the regression model correctly described the experimental data. The extraction of pigments was not significantly affected by the extraction temperature and time, similar to the findings of a previous study [39]. Unlike in previous studies [40, 41], the temperature did not affect the extraction of lipids. This phenomenon could be explained by the presence of isopropanol in this study, which helps to diffuse lipids from the cells. There was an increasing tendency for the dependent variables with increasing extraction time and solvent-to-biomass ratio. From the response surface analysis, we determined that the optimal conditions were 35.7 °C for extraction temperature, 150 min for extraction time, and the ratio of solvent to solid of 100 mL g^−1^.

### Production of macular pigment-enriched microalgal oil

To confirm the actual product, edible microalgal oil from dZA1 was produced using the optimized methods described above. dZA1 was cultivated under one-step cultivation conditions using a 1/4NP2A medium. The biomass yield of dZA1 in 1/4NP2A medium was approximately 70% that in the normal medium (Fig. 1C; Additional file 1: Fig. S1A). According to previous reports, the biomass productivity or cell growth of the starchless strain, such as dZA1, could be affected by the reduction of starch synthesis by *AGP* knockout under nitrogen-starvation conditions [14, 34, 42]. Under the same conditions, total lipid and pigment production were confirmed, and the productivity was compared to that of the *zep*. Lipid productivity of dZA1 was 131.53 ± 16.62 mg/g/day, which was 69% higher than that of *zep* (77.87 ± 8.72 mg/g/day). Pigment productivity of dZA1 (lutein plus zeaxanthin: 0.90 mg/g/day) was also 29% higher than that of *zep* (0.7 mg/g/day) (Fig. 4C, D; Additional file 1: Table S1). This result indicated that higher amount of pigment-enriched oil can be produced from dZA1 than parental strain in the optimized process.

**Fig. 6:**
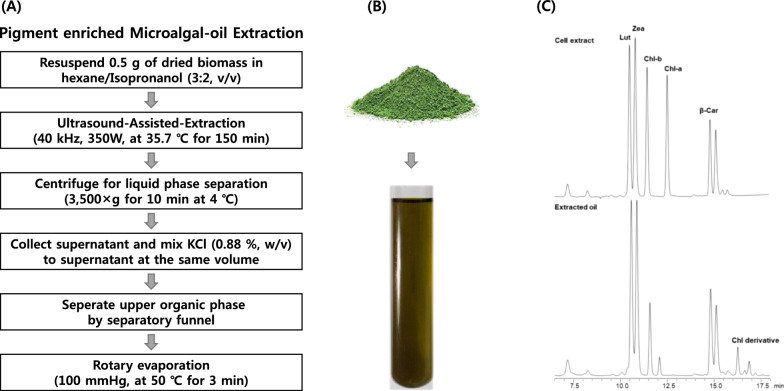
Macular pigment-enriched edible microalgal oil extraction. **A** Flow chart of the modified hexane extraction method. The method of extracting lipids and pigments simultaneously was optimized based on the Korean Food and Drug Administration Notice. **B** Pigment-enriched microalgal oil extracted from dZA1 biomass. **C** Pigments analysis by high-performance liquid chromatography in the extracted oil. The pigment profile of extracted oil (lower) was compared with the pigment profile of cell extract (upper)

To evaluate the final yield for edible use, microalgal oil was extracted by the optimized edible oil extraction method using the HI solution (Fig. 6A, B). Pigments in the extracted oils were analyzed by HPLC. The pigment-enriched oil contained large amounts of lutein and zeaxanthin (Fig. 6C). Finally, it was possible to obtain 196 ± 20.1 mg edible oil containing 8.42 ± 0.92 mg g^−1^ lutein of oil and 7.69 ± 1.03 mg g^−1^ zeaxanthin of oil from 1 g of dZA1 (Table 1). The extraction yield for edible oil obtained by the food-grade method was calculated as 37.2% and approximately 90% of the pigments was recovered in extracted oil. Thus, edible microalgal oil with highly concentrated pigments was produced, however, the extraction yield of algae oil using food-grade method needs to be improved.

**Table 1:** Edible microalgal oil production from dZA1 using the optimized process (n  = 5)

Biomass (mg L^−1^)	Total lipid (mg g^−1^ DCW)^a^	Pigments (mg g^−1^ DCW)^a^	Edible oil extract (mg g^−1^ DCW)^b^	Pigment in edible oil (mg g^−1^ oil)^b^
66.0 ± 0.03	526.1 ± 66.2	L: 1.70 ± 0.174	196 ± 20.1	L: 8.42 ± 0.918
		Z: 1.86 ± 0.094		Z: 7.69 ± 1.034

L: lutein; Z: zeaxanthin

^a^Amount of analytical extract

^b^Amount of edible oil extract

## Conclusion

We generated dZAs through CRISPR-Cas9-mediated *AGP* knockout targeting *zep* for greater oil production. For industrial feasibility, we tested the culture process and the macular pigment-enriched oil extraction method. Optimization of the medium for one-step cultivation shortened the production process by approximately 33%. Comparing the two-step and one-step processes, the oil productivity of the dZA strains in the one-step process was 1.75-fold that in the two-step process, but the macular pigment productivity was almost the same for both processes. We also developed an extraction method with hexane/isopropanol to make the oil safe for consumption. Microalgal oil contains a high content of lutein and zeaxanthin was produced from dZA1 mutant under the one-step process, and this is expected to beneficial for human eye health. This new perspective on the development of high value-added and functional microalgal oils could contribute to the commercialization of microalgae-based products.

## Method

### Microalgal strain and culture conditions

*Chlamydomonas reinhardtii* CC-4349 cw15 mt^−^ (provided by Chlamydomonas Resource Center, University of Minnesota, USA), *zep* line made by Baek et al. [24] and double knockout mutants were grown in tris acetate phosphate (TAP) medium. Cells were cultured on a shaker (120 rpm) with light intensity of 60 μmol photons m^–2^ s^–1^ at 25 °C. To produce pigment-enriched edible oil, cells grown in normal TAP medium were cultured in nitrogen-depleted medium to induce lipid production. To simplify the culture process, the culture medium was adjusted to 1/4NP2A medium. The 1/4NP2A contained 1.875 mM NH_4_Cl, 0.155 mM K_2_HPO_4_, 0.103 mM KH_2_PO_4_, and 2 mL L^–1^ glacial acetic acid; the other components were the same as that of the TAP medium.

### CRISPR-Cas9 RNP-mediated knock-out

The *C. reinhardtii zep* mutant was gene edited as described in a previous study with slight modifications [24]. ADP-glucose pyrophosphorylase (AGP) gene (Cre03.g188250.t1.2) was selected to editing target to develop a double knockout mutant producing macular pigment enriched microalgal oil. The sgRNA target sequence of AGP gene was 5ʹ-TAGCATGGCCCTGAAGATGCGGG-3ʹ. Ribonucleoprotein (RNP) complexes were generated by premixing 100 μg of Cas9 protein purchased (ToolGen, Seoul, South Korea) and 70 μg of in vitro transcribed sgRNA (GeneArt™ Precision gRNA Synthesis Kit, Invitrogen, CA, USA). Then, RNP complex was incubated 5 × 10^5^ cells for 5 min on ice and transferred into a 0.4-cm electroporation cuvette. Cells and the RNP complex mixture were pulsed using a Gene Pulser Xcell Electroporation System (Bio-Rad, CA, USA) with a set pulse at 600 V (50 µF, and 200 Ω resistance). After electroporation, the cells were plated on TAP agar plates.

### Mutant screening by iodine solution assay and genome sequence analysis

To confirm the AGP knockout mutant, liquid-cultured cells were visualized by iodine staining. Candidate mutants from the agar plates were transferred to 96-well culture plates. Transferred cells were cultured in nitrogen-deprived TAP medium for 3 days and were then stained with 1 μg of iodine to visualize starch accumulation (dark green or dark grey). The mutants without color change (green or yellowish green) were selected as *AGP* knock-out mutants, and the genomic DNA was isolated and subjected to PCR with specific primers which are adjacent to sgRNA target sites in *AGP* (F: 5ʹ-TGGGCACGACTTGCATTGTGT-3ʹ, R: 5ʹ-AATGGGCCAGCGCGAGCATA-3ʹ). The PCR products were analyzed by gel electrophoresis and by Sanger’s sequencing method (Macrogen, Seoul, South Korea). The *AGP* gene sequence of the mutants was aligned with the innate gene sequence to confirm mutation.

### Quantification of starch

The content of microalgal starch was assayed using a assay kit including amyloglucosidase/α-amylase method (Megazyme, Wicklow, Ireland). The dried biomass was fragmented with a pestle and resuspended in 80% ethanol. Thermostable α-amylase (3000 U mL^−1^) in sodium acetate buffer (100 mM sodium acetate buffer, pH 5.0) was added to each sample. The samples were incubated in a boiling water bath for 12 min, followed by heating at 50 °C. Amyloglucosidase (3300 U mL^−1^) in sodium acetate buffer was then added to each sample before heating. The samples were then kept at 50 °C for 30 min and then centrifuged at 2000×*g* for 10 min. Glucose in the supernatant was assessed using the glucose oxidase reaction based on the colorimetric method (510 nm). Total starch content was calculated according to the following formula provided by the manufacturer:

$${\text{Starch}}, \% = \Delta {\text{A}} \times \frac{{\text{F}}}{{\text{W}}} \times {\text{FV}} \times 0.9$$

ΔA: absorbance; F: glucose amount (100 µg)/absorbance of standard glucose; FV: final sample volume (mL); and W: sample weight (mg).

### Lipid analysis

To confirm the presence of lipid bodies in cells, neutral lipids were stained using BODIPY™ 505/515 (Thermo Fisher Scientific, MA, USA). Cells were mixed with 0.04 µg/mL of BODIPY using a vortex mixer, and the mixture was kept at room temperature for 5 min in the dark. BODIPY-stained lipid droplets were examined by fluorescence microscopy (Eclipse Ni, Nikon, Tokyo, Japan). The fluorescence detection wavelengths is 535 ± 23 nm for the FITC filter for BODIPY and 630 ± 30 nm with the Texas RED filter for chloroplast autofluorescence.

Total lipid contents were measured according to the method described by Bligh and Dyer [43]. The cells were collected after lipid induction and frozen. The cell pellets were resuspended in 4 mL of deionised water, and then 10 mL of methanol and 5 mL of chloroform were added sequentially in a 4:10:5 ratio. After overnight incubation in the dark at room temperature, 5 mL of deionised water and 5 mL of chloroform were added, resulting in a 9:10:10 ratio of water: methanol: chloroform. The extracts were centrifuged for 10 min at 2000×*g* to separate the two layers. The chloroform layer, which contained lipids, was transferred to a pre-weighed glass bottle and evaporated on a hot plate. The difference in the weight of the bottle before and after evaporation represented the lipid content.

To measure neutral lipids (TAG), the algal cells were stained with the Nile red method described by Chang et al. [44] with some modifications. The fluorescence of Nile red was measured using a Varioskan Flash microplate reader (Thermo Fisher Scientific, USA). The fluorescence intensity was normalized to that of the cells (100 × 10^4 ^cells) in the sample. Excitation and emission wavelengths of 530 nm and 595 nm, respectively, were selected. Data are expressed as the mean and standard deviation of three replicates.

### Pigment analysis by HPLC

Pigments in cells were extracted with 90% (v/v) acetone. The extracts were analyzed using a Shimadzu Prominence HPLC model LC-20AD (Shimadzu, Kyoto, Japan) equipped with a Spherisorb 5.0 μm ODS1 4.6 × 250 mm cartridge column (Waters, Milford, USA). The pigment was detected by a photodiode array detector at 445 nm and 670 nm absorbance, as described previously [45].

### Fatty acid analysis by gas chromatography

Fatty acids were quantified according to the method described by Garcés and Mancha [46]. Lyophilized cells were treated with 2 mL of methylation mixture (MeOH: benzene: DMP (2,2-dimethoxy-propane): H_2_SO_4_, 39: 20: 5: 2) in a 4-mL vial with a Teflon cap. Then, 1 mL of heptane was added. The mixture was incubated at 95 °C for 2 h for the extraction. After cooling at room temperature, the separated organic phase (upper phase) was collected. Pentadecanoic acid (Sigma-Aldrich, MO, USA) was used as the internal standard. Fatty acid methyl esters (FAMEs) were analyzed using a gas chromatograph (7890A GC, Agilent technology, CA, USA) equipped with a flame ionisation detector and a DB-23 column (60 m × 0.25 mm × 0.25 µm); (Agilent Technology, CA, USA). Helium was used as carrier gas. For FAME analysis, the injection volume was 1 μL (split ratio, 1:10). The injector temperature was 250 °C, and the detector temperature was 280 °C. Initially, the oven temperature was held at 50 °C for 1 min, gradually increased to 250 °C and then held at 250 °C for 5 min. This study was supported by the National Instrumentation Center for Environmental Management (NICEM).

### Lipid extraction for edible use

To extract the edible oil at low temperatures over a short time period, ultrasound-assisted extraction (UAE) was used. A 0.5 g sample of dried biomass and 50 mL of 3:2 (v/v) hexane/isopropanol mixture (HIP) was used to extract pigment-enriched oil, and the mixture was sonicated in an ultrasonic bath at a frequency of 40 kHz and power of 350 W for 150 min at 35.7 °C (Powersonics 505, Hwashin Technology, Seoul, Korea). The sonicated mixture was centrifuged at 3500×*g* for 10 min at 4 °C to separate the liquid phase. The supernatant was collected and mixed with 0.88% KCl solution of the same volume. To collect the organic phase, the supernatant with the KCl solution was transferred to a separating funnel, and the upper phase was collected. Finally, the organic solution was evaporated by rotary evaporation at 50 °C for 3 min. The final extract was mixed with acetone, filtered using a 0.2-µm syringe filter for HPLC analysis, and was mixed with chloroform for lipid analysis.

### Statistical analysis

All experiments were performed at least in triplicate. Student’s *t *test was performed to compare the quantitative values of Nile red fluorescence, starch, and lipid contents between the wild type and the mutants. The p values that are p  < 0.05 are considered to be statistically significant. Data obtained from pigment contents, total lipid productivity, and pigment productivity were analyzed by two-way ANOVA model as described by Assad [47]. Tukey’s multiple comparison was used to test the significance of differences among means. Data are expressed as mean  ±  standard deviation (SD).

## Supplementary Information

**Additional file 1: Table S1. **Biomass, total lipid, pigment production from WT, *zep*, dZA1, dZA2 from the two-step and one-step process (n = 3). **Figure S1**. Growth curves of WT, *zep,* dZA1, dZA2 from **A** one-step process (n = 6) and **B** two-step process, TAP-N medium (grey) (n = 3).

## Data Availability

All data generated or analyzed in this study are included in this article.
