# Supplementary material for: Macular pigment-enriched oil production from genome-edited microalgae
Source: Microb Cell Fact. 2022 Feb 19;21:27. doi: 10.1186/s12934-021-01736-7 (PMC8858528; doi:10.1186/s12934-021-01736-7)
Supplement: Supplementary file 1 — Additional file 1: Table S1. Biomass, total lipid, pigment production from WT, zep, dZA1, dZA2 from the two-step and one-step process (n = 3). Figure S1. Growth curves of WT, zep, dZA1, dZA2 from A one-step process (n = 6) and B two-step process, TAP-N medium (grey) (n = 3). [file 12934_2021_1736_MOESM1_ESM.pptx]

## Slide 1
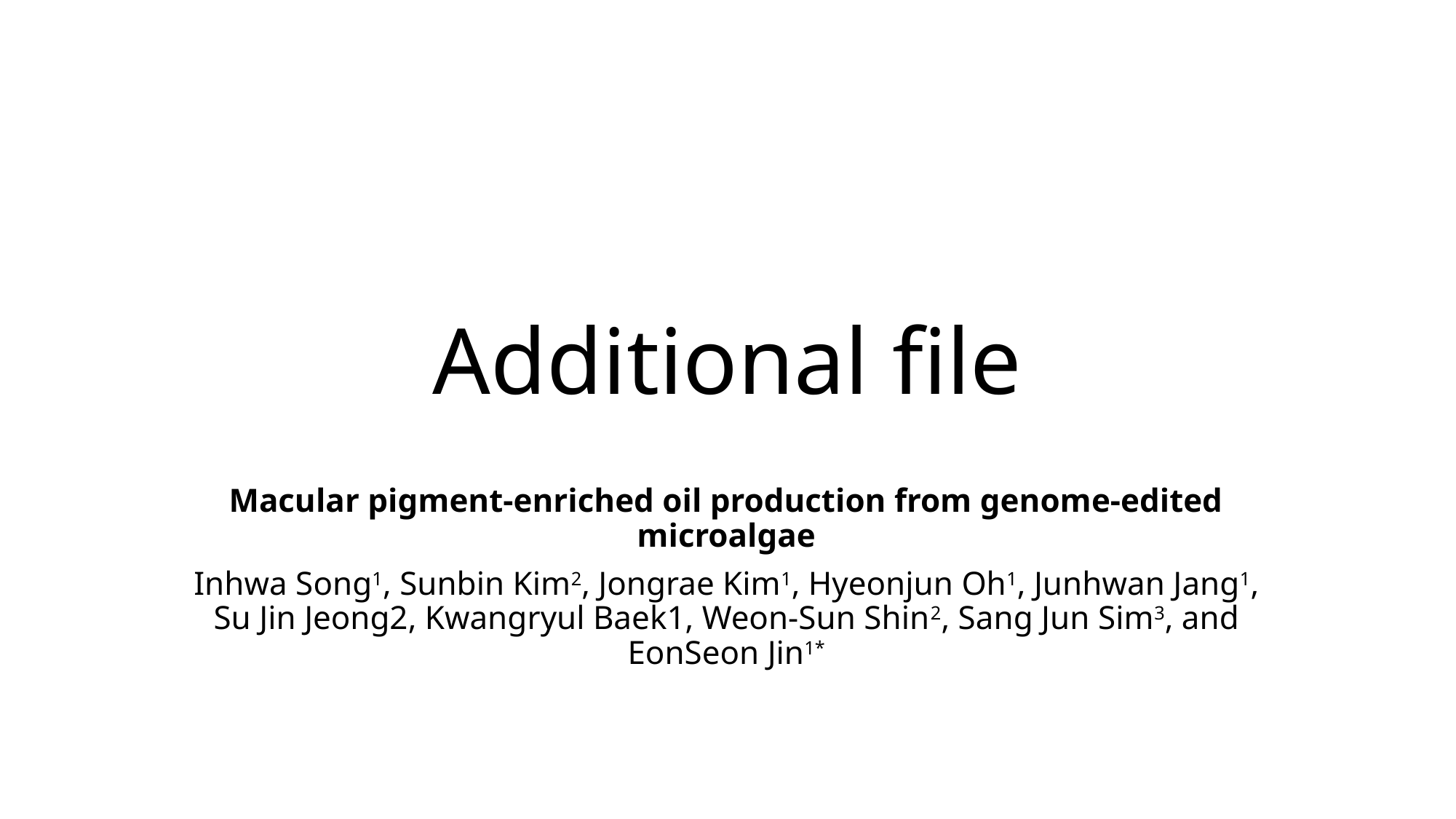

# Additional file
Macular pigment-enriched oil production from genome-edited microalgae
Inhwa Song1, Sunbin Kim2, Jongrae Kim1, Hyeonjun Oh1, Junhwan Jang1, Su Jin Jeong2, Kwangryul Baek1, Weon-Sun Shin2, Sang Jun Sim3, and EonSeon Jin1*

## Slide 2
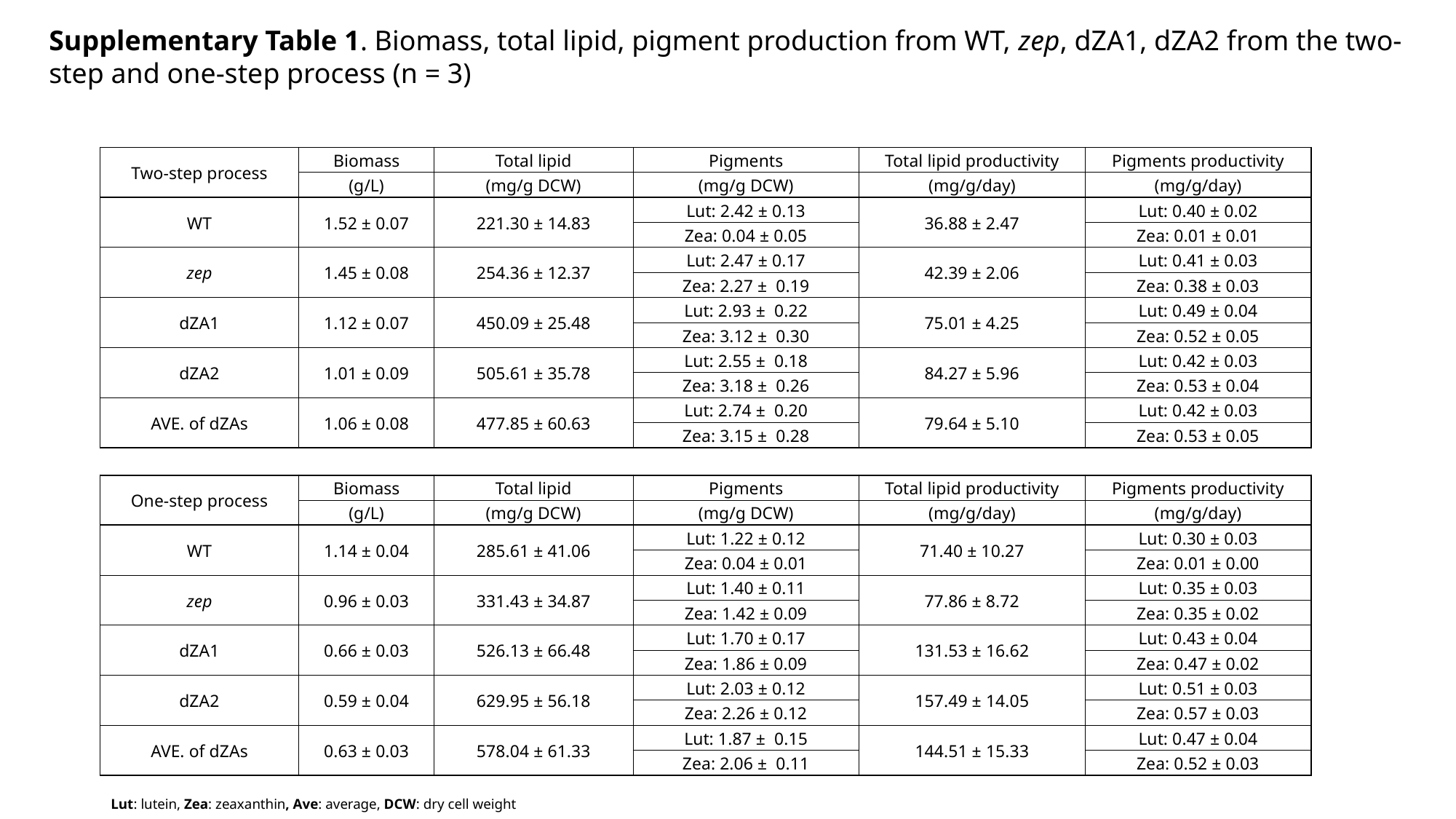

Supplementary Table 1. Biomass, total lipid, pigment production from WT, zep, dZA1, dZA2 from the two-step and one-step process (n = 3)
| Two-step process | Biomass | Total lipid | Pigments | Total lipid productivity | Pigments productivity |
| --- | --- | --- | --- | --- | --- |
| | (g/L) | (mg/g DCW) | (mg/g DCW) | (mg/g/day) | (mg/g/day) |
| WT | 1.52 ± 0.07 | 221.30 ± 14.83 | Lut: 2.42 ± 0.13 | 36.88 ± 2.47 | Lut: 0.40 ± 0.02 |
| | | | Zea: 0.04 ± 0.05 | | Zea: 0.01 ± 0.01 |
| zep | 1.45 ± 0.08 | 254.36 ± 12.37 | Lut: 2.47 ± 0.17 | 42.39 ± 2.06 | Lut: 0.41 ± 0.03 |
| | | | Zea: 2.27 ± 0.19 | | Zea: 0.38 ± 0.03 |
| dZA1 | 1.12 ± 0.07 | 450.09 ± 25.48 | Lut: 2.93 ± 0.22 | 75.01 ± 4.25 | Lut: 0.49 ± 0.04 |
| | | | Zea: 3.12 ± 0.30 | | Zea: 0.52 ± 0.05 |
| dZA2 | 1.01 ± 0.09 | 505.61 ± 35.78 | Lut: 2.55 ± 0.18 | 84.27 ± 5.96 | Lut: 0.42 ± 0.03 |
| | | | Zea: 3.18 ± 0.26 | | Zea: 0.53 ± 0.04 |
| AVE. of dZAs | 1.06 ± 0.08 | 477.85 ± 60.63 | Lut: 2.74 ± 0.20 | 79.64 ± 5.10 | Lut: 0.42 ± 0.03 |
| | | | Zea: 3.15 ± 0.28 | | Zea: 0.53 ± 0.05 |
| One-step process | Biomass | Total lipid | Pigments | Total lipid productivity | Pigments productivity |
| --- | --- | --- | --- | --- | --- |
| | (g/L) | (mg/g DCW) | (mg/g DCW) | (mg/g/day) | (mg/g/day) |
| WT | 1.14 ± 0.04 | 285.61 ± 41.06 | Lut: 1.22 ± 0.12 | 71.40 ± 10.27 | Lut: 0.30 ± 0.03 |
| | | | Zea: 0.04 ± 0.01 | | Zea: 0.01 ± 0.00 |
| zep | 0.96 ± 0.03 | 331.43 ± 34.87 | Lut: 1.40 ± 0.11 | 77.86 ± 8.72 | Lut: 0.35 ± 0.03 |
| | | | Zea: 1.42 ± 0.09 | | Zea: 0.35 ± 0.02 |
| dZA1 | 0.66 ± 0.03 | 526.13 ± 66.48 | Lut: 1.70 ± 0.17 | 131.53 ± 16.62 | Lut: 0.43 ± 0.04 |
| | | | Zea: 1.86 ± 0.09 | | Zea: 0.47 ± 0.02 |
| dZA2 | 0.59 ± 0.04 | 629.95 ± 56.18 | Lut: 2.03 ± 0.12 | 157.49 ± 14.05 | Lut: 0.51 ± 0.03 |
| | | | Zea: 2.26 ± 0.12 | | Zea: 0.57 ± 0.03 |
| AVE. of dZAs | 0.63 ± 0.03 | 578.04 ± 61.33 | Lut: 1.87 ± 0.15 | 144.51 ± 15.33 | Lut: 0.47 ± 0.04 |
| | | | Zea: 2.06 ± 0.11 | | Zea: 0.52 ± 0.03 |
Lut: lutein, Zea: zeaxanthin, Ave: average, DCW: dry cell weight

## Slide 3
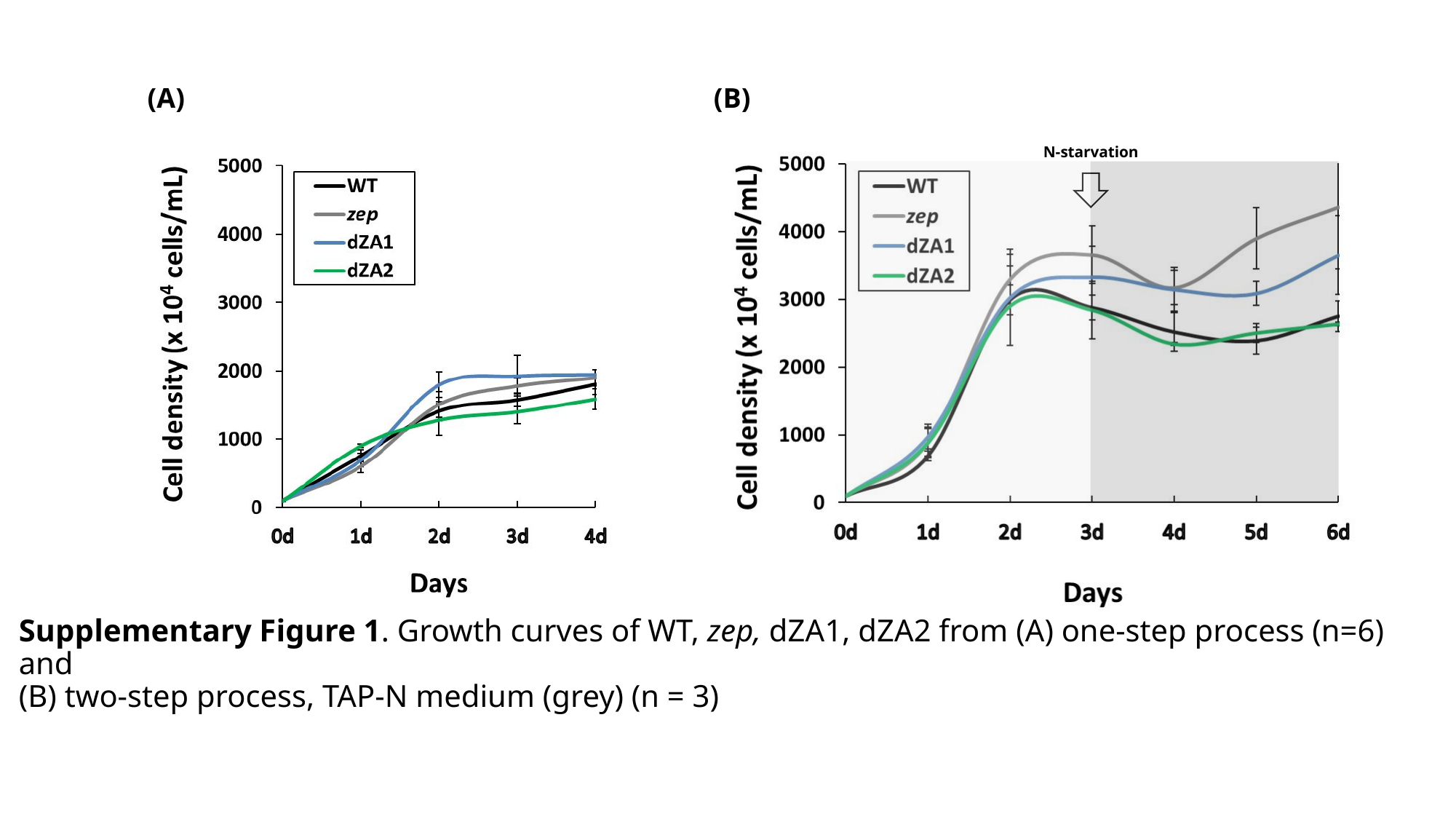

(A)
(B)
N-starvation
# Supplementary Figure 1. Growth curves of WT, zep, dZA1, dZA2 from (A) one-step process (n=6) and (B) two-step process, TAP-N medium (grey) (n = 3)
